# Supplementary material for: Optimal Expression, Function, and Immunogenicity of an HIV-1 Vaccine Derived from the Approved Ebola Vaccine, rVSV-ZEBOV
Source: Vaccines (Basel). 2023 May 12;11(5):977. doi: 10.3390/vaccines11050977 (PMC10223473; doi:10.3390/vaccines11050977)
Supplement: Supplementary file 1 [file vaccines-11-00977-s001.zip › vaccines-2362646-supplementary_proof.pdf]

File S1: Supplementary methods

Transfection.

Transfections of chimeric HIV-1 Env expression vectors were performed using polyethylenimine (Sigma Aldrich, St. Louis, MO) according to the manufacturer's instructions. The rescue of rVSV vectors was performed using Lipofectamine 2000 according to the manufacturer's instructions (ThermoFisher Scientific).

Western blot analysis.

For the analysis of the total cellular expression of chimeric HIV-1 Env proteins, 10 cm dishes containing HEK-293T cells at 70% confluency were transfected with 4 μg of chimeric HIV Env expression vectors and either 1 μg of HIV Rev expression vector (pRSV-Rev) or empty pcDNA3.1 vector. The pRSV-Rev was a gift from Didier Trono (Addgene plasmid # 12253; http://n2t.net/addgene:12253; RRID:Addgene_12253). After 24 hours, cells were washed with 1X PBS, followed by lysis (0.5 M HEPES, 1.25 M NaCl, 1 M MgCl2, 0.25 M EDTA, 0.1% Triton X- 100, 1X complete protease inhibitor; Roche, Indianapolis, IN). Cells were placed on a rotator for 20 minutes at 4°C before scraping and removing insoluble cellular debris by centrifugation at 20,000 x g for 20 minutes. Cell lysates were boiled at 93°C in 5X SDS-PAGE sample buffer (0.312 M Tris pH 6.8, 25% 2-Mercaptoethanol, 50% glycerol, 10% SDS) for 15 minutes and stored at - 20°C. Proteins were separated via 8% SDS-PAGE gel and subsequently transferred to nitrocellulose membranes. Membranes were blocked in 5% non-fat skimmed milk (BioShop Canada, Burlington, Canada) in 1X TBST, containing 0.1% Triton X-100, for 1 hour at room temperature. This was followed by an overnight incubation at 4°C with primary antibodies: clarified hybridoma supernatant containing mouse anti-gp120 (B13) monoclonal IgG (provided by George Lewis, Institute of Human Virology, Baltimore, MD, and Bruce Chesebro, NIAID, Hamilton, MT) and anti-actin mAb (1:3000; ThermoFisher Scientific). Membranes were then washed with 1X TBST and incubated for 1.5 hours with the appropriate species-specific HRP-conjugated secondary antibodies (1:3000 dilution, Thermo Scientific). All blots were developed and quantified using ECL substrates (Millipore Inc., Billerica, MA) and either a

Gel Doc EZ Imager (Bio-Rad) or C-DiGit chemiluminescence Western blot scanner (LI-COR Biosciences).

For the analysis of rVSV particle protein content, 10^6 plaque forming units (PFU) of rVSV particles were mixed with 5X SDS-PAGE sample buffer and boiled for 15 minutes at 93°C. Subsequently, the proteins were separated via 8% SDS-PAGE gel and transferred to nitrocellulosemembranes. Membranes were blocked in 1% bovine serum albumin (BSA) (in TBST containing 0.1% Triton X-100) for 1 hour at room temperature, then incubated overnight at 4°C with variousantibodies: mouse anti-gp120 (B13) mAb, mouse anti-VSV-N (10G4) mAb (1:1000; Kerafast), mouse antiZaire Ebola virus glycoprotein (4F3) mAb (1:1000; IBT Bioservices), and rabbit anti- VSV-G (1:1000; Abcam). Membranes were then washed in 1X TBST and incubated for 1.5 hourswith the appropriate species-specific HRP-conjugated antibodies (1:1000 for all; Thermo Scientific). All blots were developed and quantified using ECL substrates (Millipore Inc., Billerica,MA) and a Gel Doc EZ Imager (Bio-Rad).

Detection of anti-HIV Env Abs using enzyme-linked immunosorbent assay.

ELISA was used to assess serum anti-HIV Env antibody titers. Plates were coated with 100 ngof D7324 (anti-HIV-1 Env gp120 C5) in 50 uL PBS and incubated at 4°C overnight. The plates were washed five times with PBS containing 0.05% Tween-20, and subsequently blocked with 5% BSA in PBS for 1 hour at 37°C. 50 ng of purified JR-FL gp140 trimer protein was subsequently added to each well and incubated for 1 hour at 37°C, followed by five washes. Sample dilutions were prepared using blocking buffer and were applied at 50 uL per well in duplicates. Samples were incubated at 37°C for 1 hour. Unbound antibodies were removed by washing with wash buffer. Next, 50 μL of HRP-conjugated goat anti-mouse IgG secondary Ab (1 μg/mL) was used for the detection of anti-HIV Env IgG Abs. Assay was developed using 50 μL/well of SureBlue TMB 1-component microwell peroxidase substrate (SeraCare) and stopped after 5 minutes with 50 μL of 0.1 N H_2SO_4. Optical densities were measured using the Cytation 5 imaging reader (BioTek) at 450 nm using Gen5 software.

Assessment of neutralizing antibodies

The neutralization activity was measured using a luciferase-based assay in TZM-bl cells as described (Montefiori et al., 2001; *Standardized Assessments of Neutralizing Antibodies for HIV/AIDS Vaccine Development*, n.d.). The assays were performed with HIV-1 NL4-3 pseudotyped with the tier 1 (subtype B) SF162 and tier 1 (subtype A) Q23 (Seaman et al., 2010). Serum samples from mice were heat-inactivated to destroy the complement by incubating at 56 °C for 1 h before use. A tenfold dilution of the heat-inactivated serum did not influence the TZM-bl cell growth and as such, the initial 1:10 serum dilution was serially diluted at 1:4 and added in quadruplicate to 96- well plates containing the TZM-bl cells and an HIV-1 pseudotyped virus at a multiplicity of infection (MOI) of 0.1. The level of virus entry/infectivity was determined at 48 hrs by monitoring the luciferase activity in the cell lysate with Britelite (Perkin Elmer) using a Victor V plate reader (Perkin Elmer). The value of more than 50% reduction in relative luminescence units (RLU) when compared with the control was determined as positive.

Interferon-gamma ELISPOT assay.

HIV antigen-specific T-cell responses were measured via IFN-γ ELISpot according to the manufacturer's instructions (BD Bioscience, San Jose, California). Briefly, multiScreenHTS IP Filter Plates (Millipore, cat#MSIPS4W105) were activated as per the manufacturer's instructions and5 x 10^5 splenocytes, obtained from mice immunized by various VSV vaccines, were added to each well. Splenocytes were stimulated with 1 μg/mL of a peptide pool, comprising of 37, 15-mer peptides with 12 overlapping amino acids and spanning over C1, V1 region of gp120 (N-terminal),(AIDSreagent repository, Cat no. 9480), at a concentration of 1 ug/ml. Splenocytes were stimulatedovernight at 37°C in 5% CO_2 in RPMI 1640 complete media. The negative and positive controls were 1% DMSO in RPMI and PMA 10 ng/ml/ 500 ng ionomicynin RPMI, respectively. Plates were then extensively washed before the subsequent incubation with biotinylated anti-mouse IFN-γ Ab. Following subsequent washes, streptavidin–horseradish peroxidase (HRP) was added to eachwell, and the plates were incubated for 1 h at room temperature. The plates were finally washed 4times and IFN-γ–secreting cells were detected using AEC Chromogen (BD biosciences). The plates were then rinsed with distilled water and dried at room temperature overnight. Spots were counted with an automated AID EliSpot Reader.

Flow cytometry.

To investigate the cell-surface expression of chimeric Env proteins, HeLa cells were transfected with the various chimeric Env expression vectors as described above. Twenty-four hours post-transfection, cells were trypsinized, washed twice with 1X PBS, and fixed in 1.5% paraformaldehyde for 15 minutes. Following the fixation, cells were washed in cell-staining buffer

(Biolegend) and stained with human anti-HIV-1 gp120 monoclonal IgG (NIH Cat # 2640, B12, 1:400) antibody for 1 hour. Cells were then washed twice and stained with donkey anti-human AlexaFluor 647 mAb (1:400) for one hour, followed by two washes in cell-staining buffer. Samples were processed using a BD FACSCanto (BD Biosciences) cytometer and the geometric mean fluorescence intensity of AlexaFluor 647 (Env) was determined for live singlet HeLa cells.

REFERENCES

Montefiori, D. C., Hill, T. S., Vo, H. T. T., Walker, B. D., & Rosenberg, E. S. (2001). Neutralizing antibodies associated with viremia control in a subset of individuals after treatment of acute human immunodeficiency virus type 1 infection. *Journal of Virology*, *75*(21), 10200–10207. https://doi.org/10.1128/JVI.75.21.10200-10207.2001

Seaman, M. S., Janes, H., Hawkins, N., Grandpre, L. E., Devoy, C., Giri, A., Coffey, R. T., Harris, L., Wood, B., Daniels, M. G., Bhatacharya, T., Lapedes, A., Polonis, V. R., McCutchan, F. E., Gilbert, P. B., Self, S. G., Korber, B. T., Montefiori, D. C., & Mascola, J. R. (2010). Tiered Categorization of a Diverse Panel of HIV-1 Env Pseudoviruses for Assessment of Neutralizing Antibodies. *Journal of Virology*, *84*(3), 1439–1452. https://doi.org/10.1128/jvi.02108-09

Standardized Assessments of Neutralizing Antibodies for HIV/AIDS Vaccine Development. (n.d.). Retrieved January 16, 2023, from https://www.hiv.lanl.gov/content/nab-reference-strains/html/home.htm

Supplementary Figure 1

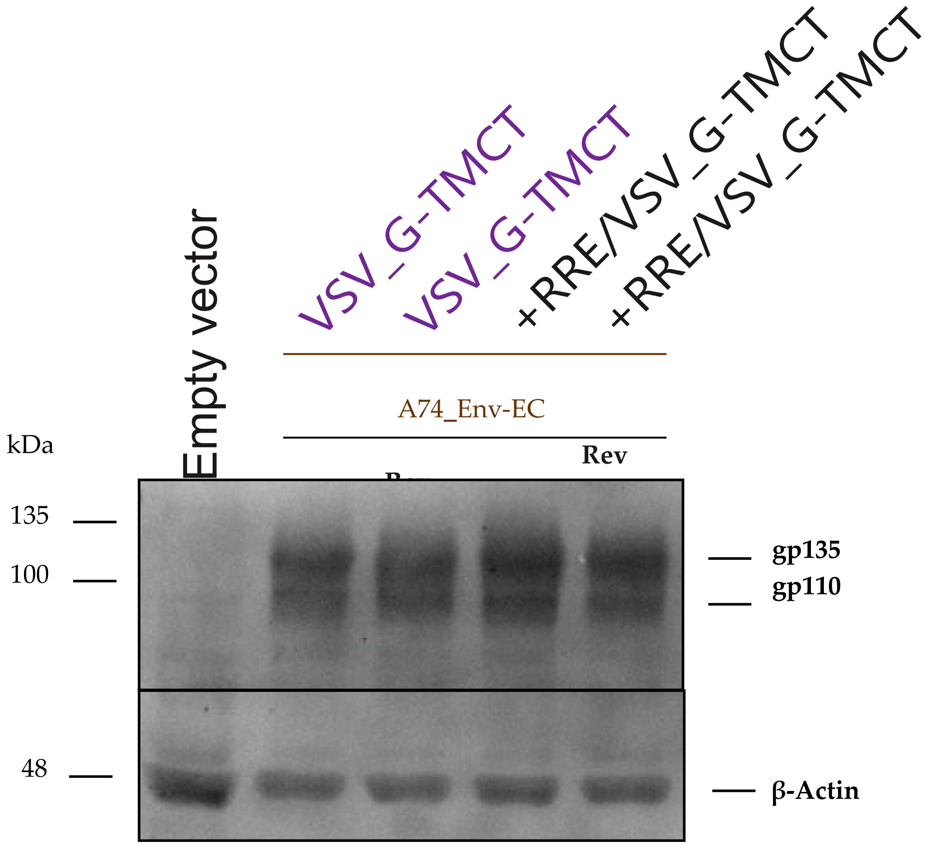

Supplementary Figure S1. (A) Schematic representation of chimeric COA74 Env proteinstested in the presence or absence of HIV-1 Rev. HEK-293T cells were transfected with chimeric Env expression vectors and twenty-four hours post-transfection were analyzed for Envexpression by western blot. (B) Western blot analysis of COA74 Env chimera expression normalized by β-Action.
